# Culpeper’s herbal *The English Physitian* and its debt to apothecary John Parkinson

**DOI:** 10.1017/mdh.2024.22

**Published:** 2024-07

**Authors:** Graeme Tobyn

**Affiliations:** School of Health, Social Work, and Sport, University of Central Lancashire, Preston, UK

**Keywords:** Nicholas Culpeper, John Parkinson, Seventeenth-century, Botanical description, Native plant medicines, The sick poor

## Abstract

In this textual comparison of seventeenth-century herbals, I show in detail that most of the descriptions and medicinal uses of English herbs included in Culpeper’s small folio *The English Physitian* (1652) and its enlargement of the following year were lifted straight out of the works of John Parkinson, apothecary. This was a deliberate act by Culpeper, to make available to the people of England the best information on native plant medicines for use in treating their illnesses. He attacked the College of Physicians of London, whom the great majority of the population could not afford to engage, for trying to keep this knowledge secret. Among later historians of the herbal tradition, Culpeper’s work was not accorded the same status as the great English herbals of William Turner, John Gerard, and John Parkinson, not because this borrowing was recognised but because its astrological content worked to divert attention from the quality and source of much of its guidance on treatment. Even contemporaries of Culpeper did not recognise the extent of the borrowing. Comparisons also reveal the limitations of Culpeper’s powers of plant description and his lack of interest in the developing science of botany. The editorial decisions Culpeper made to reduce a great folio herbal to a much smaller book to be sold for 3d touch on domestic and other non-medical uses, while points of discussion common to both authors such as the doctrine of signatures and superstitious beliefs about plants are explored.

## The English Physitian Enlarged

In 1652 Nicholas Culpeper, self-styled English physician and astrologer, published his most enduring work, a small folio herbal of 175 pages, entitled *The English Physitian; Or, an Astrologo-Physical Discourse of the Vulgar Herbs of This Nation: Being a Compleat Method of Physick.* It was a bestseller, one of several from his commercial partnership with publisher Peter Cole, which began with the immediate success of a scandalous translation of the London Pharmacopoeia Cole set him to undertake in 1647, and ended only with the early death of the author in 1654.^1^ Cole subsequently continued to append the Culpeper name to title pages of English translations of continental medical texts he had commissioned from others to maximise his sales of the brand until his own death from suicide in 1665, probably provoked by illness.^2^ The pharmacopoeia and the herbal continued in print, nevertheless, and brought profit to their new publishers into the eighteenth century.

Uniquely among Culpeper’s genuine works, *The English Physitian Enlarged* – for I will focus on the slightly expanded version issued in a smaller octavo size in 1653, on which subsequent editions were based – reflected his own knowledge as ‘a student in astrology and physick’ and his consultations with ‘Dr Reason’ and ‘Dr Experience’. A list of authors of herbals and other medical texts he made use of appeared at the front of the book, but the main text only occasionally mentioned them.^3^ The botanist, physician, and apothecary Richard Pulteney (1730–1801) generously complimented the botanical content of the work, writing in 1790, with some perspicacity as I will show, that ‘of the astrological herbalists, Nicholas Culpeper stands eminently forward. His “herbal” first printed in 1652, which continued for more than a century, to be the manual of good ladies in the country, is well known; and, to do the author justice, his descriptions of common plants were drawn up with a clearness and distinction that would not have disgraced a better pen’.^4^

Historians earlier in the twentieth century, when Culpeper was still considered a ‘quack’ rather than a medical educator, could not see past the herbal’s astrological content, which ‘infected’ seventeenth-century England with ‘astrological botany’. Agnes Arber’s *Herbals, their origin and evolution: A chapter in the history of botany, 1470-1670* (1912), a seminal work on herbals of the period, thus refused to name Culpeper alongside the great English herbalists William Turner, John Gerard, and John Parkinson.^5^ Arber’s focus was on the development of the future science of botany, whereas *The English Physitian* taught its readers how to use plants as medicines. The English herbal Culpeper considered the best of the three herbal writers, Parkinson’s *Theatrum Botanicum*, Arber rated the worst for its reversion of botany to a mere handmaid to medicine: before the existence of a rational order of plant classification, Parkinson had divided the plants in his great work into seventeen ‘tribes’, based partly on their medicinal qualities and partly on habitat.^6^ Be that as it may, Fabrizio Baldassarri in his 2023 review of plants in the sixteenth and seventeenth centuries gave credit to Arber’s recognition that ‘workers in the field of medicine lay the foundations of the copious and exact knowledge of plants’, but his much more complete review of relevant European texts of the period did not need to cite those of Parkinson.^7^ Likewise, Eleanour Sinclair Rohde’s well-known study *The Old English Herbals* (1922), which also drew the line at Parkinson and called Culpeper an ‘old rogue’ whose ‘name will always be associated with his herbal’ in which his astrological ideas were ‘a travesty rather than a reflection of the ancient astrological lore’, has been surpassed by Sarah Neville’s recent study of English herbals, which failed to mention Culpeper at all, let alone with respect to Parkinson’s herbals.^8^ Culpeper’s astrological herbal medicine ensured he remained a ‘quack’ in the eyes of historians until the 1960s when F.N.L. Poynter preliminarily concluded that Culpeper probably really did pen all the translations of leading European medical writers of his age to which his name was attached on title pages – for Culpeper had been ‘born a gentleman and brought up a scholler’ and knew his Latin – and suggested that, by providing such a comprehensive body of medical literature in the English language, he enjoyed more influence over medicine in England for the hundred years after his death than either William Harvey or Thomas Sydenham.^9^

## Borrowing from the works of John Parkinson

Modern scholarship tucked away in two unpublished doctoral theses contended that apothecary John Parkinson’s *Theatrum Botanicum* (1640) is the principal source for the herbal and Pulteney’s ‘better pen’.^10^ As with his translations and other genuine writings, reliance on a single author was Culpeper’s *modus operandi*, but the extent of the dependence of the herbal on the works of Parkinson I make plain here. Through a close textual comparison of *The English Physitian Enlarged* with Parkinson’s *Theatrum Botanicum* and his *Paradisi in Sole Paradisus Terrestris* (1629), I have calculated that Culpeper put together 92% of all his plant entries from an editing of their equivalents in these sources.^11^ How did Culpeper streamline Parkinson’s massive folio of nearly 1800 pages describing over 3800 plants known to European writers, a work he judged ‘an hundred times better’ than Gerard’s *Herball*, to an octavo of just over 300 pages containing 328 accessible English medicinal herbs?^12^ I will explore Culpeper’s choices in the editing process – what was kept, what was excised, and what was adapted and why – and what this tells us about his own botanical and medical knowledge. I intend in this way to contribute to debates about the popularisation of medicine, of which Culpeper is well known to have been the leading figure in seventeenth-century England, and to understanding the connections between natural history and medicine.

Furthermore, Culpeper wrote in a time of increasing challenge to the dominance of Galenism when commercial distillates, salts, and oils derived from Paracelsian *chymistry* were on the rise. *The English Physitian Enlarged* straddled these tensions by promoting to the public the collection and preparation of native herbs into galenical forms of medicines that countered distempered organs of the body and imbalances of temperament allopathically, as laid out in his *Galen’s Art of Physick* published the previous year, while at the same time selecting the most favourable medicaments to use following astrological indications to effect treatment by sympathy or antipathy as ‘the two hinges upon which the whole body of physick turns’.^13^

Table 1 shows that, of 328 plant entries in *The English Physitian Enlarged*, Culpeper derived 303 (92%) of them wholly or mostly from Parkinson’s writings, while twenty are Culpeper’s own original statements. Five of these are quite short and discuss a single aspect of the herb and its use: the signatures of two variants of clover Culpeper found; the use of ointments of primroses and crowfoot; and a distilled water of chives. Seven other longer entries detail the medicinal uses of garden orach (arrach), wild clary, back cresses, cucumbers, artichokes, ladies smock, and woad, including its unexpected aside on bees. The remaining eight entries are dominated by astrological explanation, including the two entries on blessed thistle and wormwood Culpeper highlighted in his address to the reader as containing ‘the key of al[l]’, the way in to understanding his system of astrological medicine.^14^ The author wove lines of ‘astrologo-physicall discourse’ that dominated the other six entries on stinking arrach, barberry, basil, heartsease, saffron, and melancholy thistle into lists of medicinal uses edited from Parkinson for such herbs as agrimony, greater celandine, cinqfoil, figwort, fumitory, lovage, and tansy, which gave these last the appearance of something completely original.

**Table 1.** Origin of entries in *The English Physitian Enlarged*

Total entries in *The English Physitian Enlarged*	Taken wholly or mostly from Parkinson’s works	Taken from Gerard’s *Herball*	Taken from Pliny	Culpeper entries	Of unknown derivation
328	303	2	1	20	2

Culpeper was certainly doing the public a favour by making available from the best English herbal a knowledge of medicinal plants cheap to buy or able to be freely gathered in fields and hedgerows at a cost of only three pence, as advertised on the small quarto’s title page, equivalent by one calculation to a price of only £2.05 today.^15^ This was achieved by omitting illustrations of the plants. Moreover, herbs such as angelica, bay, saffron, hemp, nettles, and blessed thistle had no written descriptions either, because the author knew they were familiar sights in gardens and fields – nettles, joked Culpeper, needed none since they ‘may be found by feeling in the darkest night’ – although it may surprise us today to note that dandelion and foxglove required delineation.^16^ Conversely, the inclusion of a costly engraved portrait of Culpeper for the frontispiece, probably insisted upon by the author, was a necessary device to promote the Culpeper image and brand identity, and it complemented the use of typography and page-layout to create authoritative claims to the validity of the medical information the book contained.^17^

By contrast, the cost of Parkinson’s herbal was very high. The translator of Dioscorides, John Goodyer, paid in 1640 thirty-six shillings for a copy, plus three shillings for the binding, which is equivalent to £355.50 today, more than 175 times as much.^18^ Another comparison is provided by documentation of the estate of John Webster (1611–82), whose library contained the 1640 folio valued at £2 15s and a 1681 edition of the *English Physitian Enlarged* worth 2s, more than twenty-seven times cheaper.^19^ Compared to other vernacular medical books published at the time for the lay reader, it was just a half or a quarter of the cost of those and closer to the price of a common almanac.^20^ Thus, Charles Webster identified Culpeper as the prime example of the ideological commitment to the vernacular of the post–Civil War period and three pence bought ‘the systematic exposition of the medical properties of freely available local plants [which] was regarded as the most rational basis for empirical medicine’.^21^ Roy Porter noted that from its first release, Culpeper’s herbal began to assume exemplary status as a household name evincing the vitality of, and demand for, ‘a type of medicine that won a measure of approval from the faculty while simultaneously gaining the custom of an avid book-buying public’.^22^

Culpeper’s borrowing from Parkinson was obvious to at least one person at the time. A British Library copy of Bentley’s pirated version of the *English Physitian* has a contemporary marginalium under the portrait of Culpeper, ‘this booke is collected out of Parkinson’s herball’, presumably as a reminder to future readers of the source of Culpeper’s descriptions.^23^ Among Culpeper’s rivals in print, William Coles, author of *The Art of Simpling* (1656), had a sense of the scale of this borrowing, writing of the dead author that ‘many books indeed he hath tumbled over, and transcribed as much out of them, as he thought would serve his turne, (though many times he were therein mistaken) but added very little of his own’.^24^ Furthermore, he had noticed the opinions of ‘Parkinson following Galen and Culpepper backing him, as usually he doth, be the matter right or wrong…’ without detecting the direct copying.^25^ The Edinburgh apothecary Matthew Mackail, who had spent some time in London in 1657 writing papers on matters of religion and the church, subsequently published a withering twenty-one-page critique of Culpeper’s character and writings in which he concluded that ‘Mr Culpeper’s writings are either only other men’s writings which he hath translated into English or collections of other men’s works which he hath deformed’.^26^

Three biographies of Culpeper published in the past thirty years also failed to single out Parkinson as Culpeper’s source, seemingly because the focus of each was elsewhere.^27^ Since then, my own preliminary study on Culpeper’s borrowing clarified that where Parkinson had already provided the description and medicinal uses of a garden plant in his earlier *Paradisi in Sole Paradisus Terrestris* (1629) and referred the reader there, Culpeper had recourse to that text also.^28^ Moreover, he kept Parkinson’s division of each plant entry into morphological description, habitat where found, time of flowering, and uses or ‘vertues’, but dispensed with the usually lengthy discussion of the Latin names other herbalists had given to each species in a time before Linnaean botanical classification, when confusion of species was all too likely. He thus saved himself time, paper, and ink; he only added some common names of plants with which he was familiar. Most recently, a chapter on Culpeper in Clare Fowler’s historical study celebrating the 400th anniversary of the publication of the London Pharmacopoeia in 2018 referred to ‘botanical information taken verbatim from John Parkinson’s Theatrum Botanicum’.^29^ I now proceed to the examination of that borrowing in detail here, using the enlarged version of Culpeper’s herbal.

## Botany and materia medica in the herbals

The way in which Culpeper edited his source texts demonstrates not only the debt he owed to Parkinson, in that he borrowed every competent plant description from him, but also his own predominant interest in the medicinal uses of herbs and not their botanical morphology and classification. The following example of the plant tutsan (Table 2) has been chosen because it is typical and short, avoiding the tedium of a lengthy example of similarity concerning a more prominent medicinal herb where nevertheless the same manner of editing pertains. Further worked examples can be read in my earlier study.^30^

**Table 2.** Example tutsan (*Hypericum androsaemum*) physical description

**Parkinson (1640) pp.575–77** *Androsaemum vulgare* Common Tutsan, or Parke leaves	**Culpeper (1656) p.363** Tutsan, *or* Park leaves
Our Tutsan hath not square but brownish shining round stalkes, crested all the length thereof, rising to be two, or sometimes three foote high, branching forth even from the bottome, but more thinly set or farther asunder, having divers joynts, and at each of them two faire large leaves standing, but more thinly set than of the other sorts, which are of a darke blewish greene colour on the upper side, and of a yellower greene underneath, turning reddish towards Autumne, but abiding on the branches all the winter. At the topes of the stalkes and branches stand larger yellow flowers than in any of the former sorts, and heads with seede likewise larger, which being greene at the first, and afterwards reddish, turn to be of a blackish purple colour, when they are through ripe, with small brownish seede within them, and then yield a reddish juice or liquor, of a reasonable good scent, somewhat resinous and of a harsh or stipticke taste, as the leaves also and the flowers bee, although much lesse, but doe not yield such a clear Claret wine liquor, as Gerard following Dodonaeus therein saith it hath. The root is brownish, somewhat great hard and wooddy, spreading well in the ground.	It hath many brownish shining round stalks, crested al the length thereof, rising to be two and sometimes three foot high, branching forth even from the bottom, having divers joynts, and at each of them two fair large leaves standing, of a dark blewish green colour on the upper side, and of a yellowish green underneath, turning reddish towards Autumn, but abiding on the branches al the winter. At the tops of the stalks and branches stand large yellow flowers, and heads with seed, which being greenish at the first, and afterwards reddish, turn to be of a blackish purple colour, when they are through ripe, with small brownish seed within them, and then yield a reddish juyce or liquor, of a reasonable good scent, somewhat resinous and of a harsh and stiptitch taste, as the leaves also and the flowers be, although much less, but do not yield such a clear Claret wine liquor, as some say it doth. The root is brownish, somewhat great, hard and woody, spreading wel in the ground.

It is evident that Culpeper copied Parkinson’s words exactly, including the same depictions of the colour of the upper and lower leaves and how they change in autumn, and the colour and quality of the juice of the plant. Parkinson’s work was indeed worthy of copying because he had imbibed the best writings of the period on plant description, nomenclature, and classification by Gaspard Bauhin and Charles de L’Obel, as well as had familiarity with the encyclopaedic three-volume herbal *Historia Plantarum Universalis* compiled by Gaspard’s brother Jean Bauhin.^31^ Culpeper could omit Parkinson’s references to other species discussed in the chapter of the *Theatrum Botanicum* (‘more thinly set than of the other sorts’; ‘larger…than in any of the former sorts’) because he was portraying only the native herb in the *English Physitian Enlarged.* When Parkinson had classified under one plant name two or three native species, Culpeper duly included these. There were forty-three entries of this kind of multiple entry, which included descriptions and uses of each species, except where one or other fell into his category of ‘too well known to need description’. In one instance only, Culpeper improved on the botanical information in the *Theatrum Botanicum*: Parkinson had identified that freshwater soldier or ‘crab’s claws’ could only be found across the channel in Germany, the Low Countries, and Italy, but Culpeper read of the botanist and apothecary Thomas Johnson finding it growing plentifully in ditches about Rotsea, a small village in Holderness above Hull, and in the Lincolnshire fens in his 1633 correction and revision of Gerard’s *Herball.* ^32^ Culpeper now included it in his work, with Johnson’s locations, while Parkinson seems not to have made the same discovery.^33^

In copying Parkinson’s description of tutsan, Culpeper also economised on the words of his source by leaving out references to other authors, as with Gerard and Rembert Dodoens in the example above, by moving them to the ‘list of authors made use of in this treatise’ in the preface. This gave the impression that Culpeper himself had researched the writings of those herbalists specifically for this work, while acting to disguise his reliance on one writer above all others. Sometimes, but for no obvious or deliberate reason, the name of a quoted author was kept in the copying, which only reinforced that impression. Issues of scholarly attribution were not so pressing at this time, not just because publishers, not authors, owned the works they published, or because censorship was suspended, but significantly because of the rampant piracy of works – two pirated versions of *The English Physitian* were issued before Culpeper and Cole issued the enlarged version, partly to discredit these imitations as deficient – which raised issues of adulteration and the correct representation of the composition of a medicine in *materia medica* texts, and which affected authority in the field.^34^ Culpeper was certainly well read in the art of physic: astrologer John Gadbury reported that Culpeper had dedicated himself to the study of medicine from 1640, around the time he left off his apothecary training.^35^ I suggest that he would have been very familiar with Gerard’s *Herball* and Lyte’s translation of Dodoens, although the tomes may not have been to hand on his bookshelves because ‘he had not many books, but those that he had were well selected’.^36^

The physical descriptions of plants in *The English Physitian Enlarged* facilitated for Culpeper’s readership the identification of useful medicinal herbs in field, hedgerow, or garden, and, except in five instances, all are Parkinson’s words, edited slightly where a few words could be saved, as shown in the example above. However, Culpeper needed to keep down costs if the work was to cost thruppence, which meant restricting its size ‘lest my book grow too big’.^37^ He justified the exclusion of the depiction of nearly one hundred plants in the expanded herbal by his intention from the beginning ‘to teach my countrymen what they know not, rather than to tell them again of that which is generally known before’.^38^ Parkinson’s purpose on the other hand had been that more expected of a herbalist, commenting for instance in his entry on the white lily that it ‘scarce needeth any description, it is so well knowne, and so frequent in every garden, but to say somewhat thereof, as I use to doe of every thing, be it never so common or knowne’.^39^ His descriptions usually followed the order of stalk, leaf, flower, fruit/seed, and root, with mention of a distinctive smell or occasionally taste, if present. Culpeper tried this, too, in his new entry on Amaranthus for the *English Physitian Enlarged*: ‘It being a garden flower, and wel known to every one that keeps of it, I might forbear the description, yet notwithstanding because some desire it, I shal give it’.^40^ Culpeper may have been responding again to feedback from readers of the first edition of his herbal by including Amaranthus. Here he wanted to describe a red amaranth or flower-gentle, which he knew at least from the florists’ shops, but found Parkinson’s main description was of a small purple variety. Unable to extract a word-for-word description, he fashioned his own from the vocabulary of his source text: small branches, tufts instead of flowers, which produced a juice of the same colour when bruised, shining black seed, the stems keeping their beauty a long time after gathering.^41^

By contrast, three of Culpeper’s four original plant descriptions, which appeared first in the *English Physitian Enlarged*, are inadequate for purposes of botanical identification.^42^ It is difficult to know which species is being referred to as ‘Back-cresses’: Parkinson’s index contains no such entry, even if the name is a misprint for ‘black cress’, and the description of leaves ‘deeply cut and jagged on both sides’, small, yellow flowers and a small stalk tough enough to ‘twist them round as you may a willow before they break’ is not specific enough. Wild clary ‘is like the other clary, but lesser…he that knows the common clary cannot be ignorant of this’, but the sparse detail provided does not match Parkinson’s description of wild clary. With orchids, ‘to describe al the several sorts of it were an endless piece of work, therefore I shal only describe the roots’, but it was more a comment on the medicinal use of the inner chamber of their double roots to provoke lust when rounded and increasing in size, and to suppress it when lank and withering. From these examples, it appears that Culpeper lacked fluency in the vocabulary of plant morphology. He referred in the preface of the herbal to his knowledge of native simples ‘most of which I knew by sight before’, which was learned during his childhood in Sussex; or, perhaps less likely, from the botanising trips led by a master of the Society of Apothecaries that as an apprentice he was required to attend in the later 1630s, perhaps even from the tutelage of expert botanist Thomas Johnson himself.^43^ Nevertheless, his contemporary and competing author on simples, William Coles, had no compunction in writing in 1656 that Culpeper was ‘a man very ignorant in the forme of simples’.^44^ This was because Culpeper’s interest lay in plants’ medicinal uses; botanical descriptions were needed to help those not familiar with certain common or garden herbs to find them.

Culpeper’s frustration with the requirements of botanical description is evident where he crudely abbreviated Parkinson’s careful delineations of form, as with the descriptions of kinds of pellitory of Spain, or when faced with twenty folio pages of crowfoots in the *Theatrum Botanicum*, divided into three classes in which Parkinson did not clearly distinguish all the native species from the foreign. Culpeper opted to describe one kind of field crowfoot himself, adding many common names including ‘gold knobs, gold cups…and butter-flowers’ and again fashioning his own version seemingly from Parkinson’s description of the ‘common upright field crowfoote’:

*‘*Abundant are the sorts of this herb, that to describe them al would tire the patience even of Socrates himself; but because I have not yet attained to the spirit of Socrates, I shal but describe the most usual.

Descript.] The most common crowfoot hath many dark leaves cut into divers parts, in tast biting and sharp, biting and blistering the tongue, it bears many flowers, and those of a bright, resplendent yellow colour, I do not remember that ever I saw any thing yellower. Virgins in ancient time used to make powder of them to strew bride-beds. After which flowers come small heads of seeds, round but rugged like a pine apple’.^45^

The morphological description is deficient once more, but the common names supplied aided identification for readers through recognition, as it does for us today when we know that the herb is a buttercup.

The other apparently original description in *The English Physitian Enlarged*, that of ferns, is even more limited: the female fern grew higher than the male but the leaves were smaller, more divided, but of an equally strong smell. The uses of male and female fern were alike ‘and therefore I shal not trouble you with any further description or distinction of them’.^46^ Culpeper was not motivated to painstakingly describe variations in morphology among related species of plants to construct a botanical knowledge of their forms. The depictions are basic – here, a plant with many leaves cut in some way, and many yellow flowers and rough or rugged seed – but for the purpose of finding a medicinally useful plant to gather, the identification is aided by sensory data from taste, smell, vision, and touch: the sharp taste, the blistering effect on tissue, the impression of colour. Such organoleptic tests are what early apothecaries and physicians had to rely on for centuries to aid identification before the formal tests and morphological descriptions of pharmacopoeias were developed.^47^ John Riddle has shown how tests for adulteration were already well developed by the time of Dioscorides and the majority of the tests recorded in his *De materia medica* were organoleptic.^48^ Culpeper’s descriptions, therefore, no matter how crude, are not of the herbalist as early botanist but of the herbalist as gatherer of plants for medicines. Parkinson had organised the ordering of plants in the *Theatrum Botanicum* partly on habitat, but partly on medicinal qualities as well.^49^ As with other writers of learned herbals, he was seeking a rational system of categorisation of plants into families, but he included much other information, including organoleptic factors. He emphasised the sharp taste and irritant effect on the tongue of various kinds of crowfoot, and when we read in the *English Physitian Enlarged* of, for instance, the hot, sharp taste of pepperwort, or the unpleasant, bitter taste and strong, heady smell of poppy sap, we are reading the organoleptic evaluation of the old herbalist dutifully passed on by his copier, Culpeper.

The visual aspect of a herb aiding both identification and an understanding of medicinal use within the parameters of a doctrine of signatures was very popular in the mid-seventeenth century and both authors accommodated it.^50^ Culpeper employed the words ‘signature’ or ‘icon’ or ‘image’ when discussing the uses of red Amaranthus flowers to stop menstrual blood flow in women (‘and so do almost al other red things’), and the small, knobbly roots of pilewort (lesser celandine) to treat haemorrhoids.^51^ The plausible view today is that the doctrine of signatures was a visual mnemonic to help recall the medicinal use of a plant learned by trial and error, but Culpeper had it the other way round: he wondered how the virtues of herbs first came to be known if not by their signatures.^52^ Consequently, Culpeper’s ‘icons’ included not only colours but also, for instance, the location where the herb liked to grow (stinking arrach and wormwood) or when its flowers opened with the sun (e.g. centaury).^53^ He believed that such signs in the elemental world were God-given to those who had eyes to see, as Adam had done in the Garden of Eden, and this was a way for Culpeper to quickly instruct his readers how to recognise what some herbs were good for. As Louis Kelly put it, Culpeper was trying to train his public in pragmatic observation, experience, and even common sense to get past the need for poring over books or running to authority. On the principle that God helps those who help themselves, divine illumination as well as good health would necessarily follow.^54^ Culpeper took one of his examples of signatures straight from Parkinson. Thus, white archangel ‘which country people vulgarly [i.e. commonly] know by the name of dead nettles’ was for the ‘whites’ (leucorrhoea), red archangel for excessive or abnormal menstrual bleeding (menorrhagia or metrorrhagia), and yellow archangel for corrupt (purulent) sores and ulcers.^55^ Among the docks, which Parkinson seems not to have considered a bearer of signatures but rather to be differentiated by their manifest degrees of cooling and drying, Culpeper added that red dock, also called bloodwort, ‘cleanseth the blood and strengthens the liver’ while yellow dock root ‘is best to be taken when either the blood or liver is afflicted by choler’ (yellow bile).^56^ Eighteen entries in *The English Physitian Enlarged* featured signatures, if Culpeper’s wider definition is allowed.

Two further clear instances of signatures in Culpeper’s herbal, which first appeared as new entries in the *English Physitian Enlarged*, featured two ‘remarkable’ types of trefoil not described by Parkinson among the thirty sorts of white and red varieties of clover in his book: the heart-trefoyl, because ‘the leaf is triangular like the heart of a man, but also each leaf contains the perfect icon of a heart, and that in its proper color, viz. a flesh colour’, which he dedicated to the sun; and the pearly-trefoyl, which differed from the common sort of clover in only one particular, that of a white spot in the middle of the leaf like a pearl, was thus a herb of the moon.^57^ Culpeper had seen how the first ‘groweth in a field between Longford and Bow, as also beyond Southwark towards Croyden’, parts of London that Culpeper was familiar with. Today the genus *Trifolium* is known to include 245 recognised species, among which leaf markings are widespread and are of either a red or a white colour that vary in position, size, and intensity.^58^ This might explain the appearance of the plants Culpeper found remarkable in their singularity but about which Parkinson, in his broader botanical overview, found nothing special.

Turning now to where herbs like to grow, the entries shown in Table 3 on the place where tutsan was found and the time of flowering are the same as in Parkinson’s text, aside from the typographical errors of wild for weald, the spelling of Rayleigh in Essex and removal of ‘later than any of the other’ regarding the time of flowering (because Culpeper was describing only the native plant). In practically all entries, the location and time of flowering are copied in a similar way. For crowfoot, Culpeper side-stepped the problem of locating where his exemplar for the many species might be found growing with humour: ‘they grow very common every where, unless you run your head into a hedg you cannot but see some of them where ever you walk’.^59^ Sometimes Culpeper added the Sussex name for a plant or its location: he recalled seeing black alders by the side of a brook in a wood called the Old Park in Barcombe, a village in that county near his childhood home of Isfield.^60^ Otherwise, his familiarity was with fields, hedgerows, and woods in London, and in the counties of Middlesex and Essex, where it bordered his home in Spitalfields, and possibly north Kent, if the path taken by Thomas Johnson and his fellow apothecary-botanists in his first published itinerary was any indication of where herborising trips headed when Culpeper was an apprentice apothecary in the 1630s.^61^ Thus, he noted seeing for himself wild clary ‘in the fields neer Grayes-Inn and…Chelsy’ and tormentil ‘almost in every broom field in Essex’.^62^

**Table 3.** Example tutsan, place and time of flowering

Common tutsan (Parkinson)	Tutsan (Culpeper)
**The Place** The first groweth in many woods, groves and wooddy grounds, as parkes and forrests, and by hedge sides in many places of this land, as in Hampsted wood, by Raily in Essex, in the wealde of Kent, and many other places needless to recite.	**Place** It groweth in many woods, groves and woody grounds, as parks and forrests, and by hedg sides in many places of this land, as in Hampsted wood, by Ratley in Essex, in the wild of Kent, and in many other places needless to recite.
**The Time** They all flower later than S.Johns wort, or S.Peters wort, and they last later than any of the other.	**The Time** It flowereth later than S.Johns, or S.Peters wort.

## Comparing virtues and uses of herbs

Having established that Culpeper was interested predominantly in the medicinal uses of English herbs rather than their botanical classification, I now examine how Culpeper extracted those uses from Parkinson’s works.

In the example in Table 4, Culpeper transferred wholesale to his herbal Parkinson’s medicinal uses of tutsan, with a qualification of the herb’s planetary ruler Saturn, to which he added an astro-medical indication for syphilitic rash. The herb performed this effect by antipathy of its ruler to Venus, the source of ‘venereal’ diseases. By contrast, Culpeper omitted tutsan’s galenic qualities and actions, stated by Parkinson at the top of his entry, because he intended all the herbs in the *English Physitian* to be used astrologically by sympathy or antipathy, and not according to whether they heated, cooled, dried, or moistened the body, or stimulated or repressed a movement of the blood or a natural evacuation of fluids. This did not deter him, however, from appending *A Key to Galen and Hypocrates, their Method of Physick* to the third edition of his translation of the London Pharmacopoeia in 1651: the important issue was to approach the translation of the works of such authorities critically and not to treat the text as sacrosanct, for the basis of the Puritan translation tradition which Culpeper exemplified was the belief that all writing that sought truth shared with the Bible the distinction of being the “word of God”.^63^ Culpeper struck his own balance between the divine and the human in the *English Physitian Enlarged* by amalgamating his critical reading of the medical tradition with his own assignation of astrological rulership over each herb. As promised in its preface, Culpeper indeed ‘drew out all the vertues of vulgar Herbs, Plants, and Trees, &c. out of the best and most approved authours I had or could get’, then he connected them with the harmony of God’s creation via astrology.^64^

**Table 4.** Example tutsan, its virtues or uses

Common tutsan (Parkinson)	Tutsan (Culpeper)
**The vertues** Tutsan moderately heateth and dryeth, yet the seede hath an abstersive qualitie, whereby it purgeth choloricke humours as St. Peters wort is said to doe, for therein and in all other things it makes the same effect, both to helpe the sciatica and goute and to heal burnings by fire. It stayeth also the bleeding of wounds, if either the greene herbs be bruised, or the powder of the dry be applied thereto. It is, and so hath formerly in all ages been accounted a soveraigne herbe to heal any wound or sore, eyther outwardly or inwardly as the name importeth; and therefore it was always one of their singular good herbes wherewith they made wound drinkes or lotions, balmes, oyles or oyntments, for any sort of greene wound, or old ulcers and sores, in all which the continual experience of many ages, to bee admirable good, hath confirmed the use thereof to be accepted, although it be not so much in request and use as formerly it was, when as chirurgions and leeches did more addict themselves to use herbes than now they doe.	**Government and vertues** It is an herb of Saturn, and a most noble Antivenerian. Tutsan purgeth chollerick humors as St. Peters wort is said to do, for therein it worketh the same effects, both to helpe the sciatica and gout and to heal burnings by fire. It stayeth also the bleeding of wounds, if either the green herbs be bruised, or the powder of the dry be applied thereto. It hath been accounted, and certainly is, a sovereign herb to heal any wound or sore, either outwardly or inwardly, and therefore always used in drinks, lotions, balms, oyles, oyntments, for any sort of green wound, or old ulcers or sores, in al which the continual experience of former ages hath confirmed the use thereof to be admirable good, though it be not so much in use now as when physicians and chirurgions were so wise as to use herbs than now they do.

Here and there, buried in the text, are a few examples of Culpeper responding directly to his source. Parkinson asserted, for instance, that syrup of orpine ‘is seldom used in inward medicines with us’ but that orpine leaf could be applied topically to the throat for quinsy; Culpeper in his entry retorted ‘let my author say what he will’, a spoonful or two of orpine syrup might be taken for quinsy and it was more pleasant and a speedier cure than the ‘dog’s turd which is the learned College’s vulgar cure’!^65^ For poisonous hemlock, Parkinson had advised that the herb could safely be applied topically to inflammations, tumours, and swellings, but not, as some advocated, to the male genitalia in cases of venereal disease, nor to women’s breasts to curtail their swelling and repress their milk, ‘by reason the places are so tender and full of vitall spirits, it often proveth that the remedy is more dangerous then the disease’.^66^ Culpeper opened on the uses of hemlock in his book: ‘Saturn claims dominion over this herb; yet I wonder why it may not be applied to the privities in a priapismus, or continual standing of the yard, it being very beneficial for that disease’. He then teasingly speculated on the astrological thinking of his source: ‘I suppose my authors judgment was first upon the opposite disposition of Saturn to Venus in those faculties and therefore he forbid the applying of it to those parts that it might not cause barrenness, or spoil the spirit procreative, which if it do, yet applied to the privities it stops lustful thoughts’.^67^ Culpeper seems to have judged that the urgency for treatment for priapism by the topical application of poisonous hemlock outweighed the potential damage to a man’s procreative spirit.

In the entry on fluellen/fluellin or lluellin, Culpeper took an oblique line from his author to mount an attack on the College of Physicians. Once again, the descriptions of two sorts of fluellin (Parkinson’s third sort, which only differed in the colour of the flowers, was omitted), their locations, and time of flowering were the same in both herbals, including the exact locations of Southfleet in Kent and three places in Huntingdonshire mentioned by Parkinson where the herb had been identified. The virtues of the herbs in each entry were also the same actions and uses, and as usual in the same order. Parkinson added a story, as a witness to the efficacy of the herb, of a man whose nose was being eaten away by a canker. His doctors appointed a surgeon to cut off the remnant of his nose to preserve the rest of the body, but a simple barber, overhearing the order, asked if he could be given a little time to try to save the nose by a use of fluellin, which his master had taught him. The juice and decoction of the herb was taken internally and the herb applied externally and the nose saved and the body returned to health. Parkinson commented that

This occasion doth make me thinke, that not onely in this herbe, but in many other simple herbes, our forefathers found helpe of many diseases, and therefore used fewer compounds: and were we in these times as industrious, to search into the secrets of the nature of herbes, as the former ages were, and to make tryall of them, we should no doubt finde the force of simples, many times no lesse effectuall than of compounds: but of this enough, yet not too much, for as I might provoke some learned to bee more industrious, and not like droanes onely to sucke the honey from others hives.^68^

Culpeper had alighted on an opinion close to his heart, that of the benefits of native simples versus the compound medicines prescribed by physicians, and the need to inform people of their uses instead of hiding them as the College of Physicians’ Latin pharmacopoeia did. His own entry continued:

Bees are industrious and go abroad to gather honey from each plant and flower, but drones lie at home and eat up what the bees have taken pains for; just so do the Colledg of Physitians lie at home, and domineer, and suck out the sweetness of other men’s labours and studies, them selves being as ignorant in the knowledge of herbs as a child of four years old, as I can make appear to any rational man by their last Dispensatory, now then to hide their ignorance, there is no readier way in the world, than to hide knowledg from their countrymen, that so no body might be able so much as to smel out their ignorance, when simples were more in use, mens bodies were better in health by far than now they are, or shall be if the Colledg can help it.^69^

Here is Culpeper’s central criticism of the College of Physicians: that from the sick who were unable to afford their attendance, the doctors hid the knowledge of how they might help themselves using native plant medicines. Only at the end of the entry did Culpeper turn to the recounted cure of the patient (a Welshman, although he did not glean this from Parkinson’s story – it may have been a familiar tale and the plant gained the variant name lluellin as a joke), and to its use for virulent sores and ‘ulcers of the French pox’ (syphilis).^70^

Other examples where Culpeper responds directly to Parkinson, as if in conversation with his author and source, can be found. Parkinson had recounted Hieronymous Bock’s recipe for preparing bittersweet (Culpeper’s amara-dulcis) to open obstructions of the liver and spleen in jaundice and complained that ‘so often as I have given it by appointment, I have knowne it to purge very churlishly’. Culpeper approved the use, because it ‘purgeth the body very gently, and not churlishly as some hold’.^71^ Of mouse-ear, Parkinson poked fun at ‘the old All-go-misse, I should say alchemists, did much commend the juice of this herbe, that it would congeale and fix mercury, but all these fancies are in these times quite dispersed and driven away, I thinke’. Culpeper thought he had missed the point, writing ‘the moon owns this herb also, and though authors cry out upon alchymists for attempting to fix quicksilver by this herb and moonwort, a Roman would not have judged a thing by the success: if it be to be fixed at al, ‘tis by lunar influence’.^72^ Such examples conjure up the image of Culpeper at home with his amanuensis, reading each section in the *Theatrum Botanicum* on a native herb and dictating the words to be written on it for the *English Physitian*, sometimes challenging an assertion or striking out an account, sometimes adding his own medical observation or simple use for a herb, always supplying an astrological assignation but keeping the entry brief.^73^

With regard to Parkinson’s large tome, the *Theatrum Botanicum*, Rebecca Laroche contrasted this ‘manlike worke of herbs and plants’ dedicated to King Charles with his *Paradisus in Sole Paradisus Terrestris*, a gardening book dedicated to the queen consort Henrietta Maria firmly rooted in a female world of tending flowers and herbs for ‘delight’ only, which lay outside the central tripartite structure of early modern medicine.^74^ This gendered comparison is ironic because most early modern people would have had experience by adulthood of being cared for and cured by a woman, to say nothing of the elderly and the sick, although women were excluded from formal medical training of any sort and, as a consequence, their healing work is barely represented in the historical record.^75^ However, it was not only in the reading and commenting on herbal and other texts of mainstream male medicine where exchange of information on healing took place. Aristocratic women produced manuscript books gathered from herbals and other sources that were sometimes published in the 1650s and beyond, while many exchanged single recipes as gifts in a form of patronage or simply from good will.^76^ Culpeper acknowledged this in his first publication, defending his translation of the physicians’ pharmacopoeia into English from accusations of encouraging unsafe practice by writing, ‘All the nation are already physitians. If you ayl any thing, everyone you meet, whether man or woman, will prescribe you a medicine for it’.^77^ In this regard, Culpeper was publishing learned knowledge about remedies cheaply, and more reliably, he believed, than many a mountebank hawking remedies at a town fair.^78^

## Culpeper on non-medical uses, issues of safety, and superstitions

So, what of Parkinson’s was omitted? Culpeper excised all uses of herbs in farriery, animal husbandry, and domestic employments, such as gentian root for “the bottes and wormes” in cattle, coltsfoot for tinder, and horsetail for scouring pewter and brass.^79^ Woad and bilberries were not mentioned as dyes for cloth or that pimpernel could be made into a cosmetic for cleansing the skin or that parsley freshened the breath. Republican Culpeper had no interest in telling his readers that the favourite strewing herb of Elizabeth I was meadowsweet. Of two recipes for tormentil cakes, Culpeper repeated the more basic one. Parkinson’s discussions of differences of opinion among his sources on the qualities and actions of a herb; or on which part of the plant had the strongest effect – seed, leaf, or root; or his correction of an author who has mis-translated a Greek term in Dioscorides were expunged to leave that which Culpeper agreed was the more correct view of the herb’s effects, the part to be used or the correct translation. The same was true of unnecessary treatments for the sting of a scorpion or the bite of a huntsman spider (*Phalangium*) – hardly likely in England and which Parkinson had taken from continental herbals – although the European viper was correctly seen ‘to be no other than our English adder’ and the poultice of wheat bran and vinegar to counter its bite included.^80^

Further elisions reflect a more ‘ad hoc’ approach as Culpeper worked through Parkinson’s text. He had originally stated that his intention was ‘to expresse my self, in such a language as might be understood by al’; and in pursuit of a plain style Culpeper revealed the influence of Francis Bacon and adherence to the principle of the Roman philosopher Seneca the Younger that ‘the language of truth is simple’ and that a polished style is morally dishonest.^81^ Indeed, in *The Epitaph* to him in *Culpeper’s School of Physick* (1659), a final collection of his unpublished notes furnished by his widow Alice and issued by Nathaniel Brook, the dead herbalist ‘had not onely thus practised Seneca, but out-stript the philosopher’.^82^ It is understandable, therefore, that Culpeper substituted difficult medical terms in the source text, such as ‘repercussive’, ‘uvula’ and ‘alexipharmacon’, with simple paraphrases in *The English Physitian Enlarged*, although this was not completely adhered to.^83^

Culpeper routinely omitted from his herbal compound medicines described by Parkinson, but one or two remained, such as Galen’s ‘powder called Diacalaminthes, and the compound syrup of calamint (which are to be had at the apothecaries)’. These mixtures required a health warning because of a potential abortifacient action, particularly if savin, a bushy Eurasian juniper ‘nursed up in almost every garden’ was included, and about these mixtures Culpeper warned ‘let not women be too busie with it, for it works very violently upon the feminine parts’.^84^ At least, it might be argued, the especially dangerous ingredient was properly compounded following an official formula, whereas Parkinson’s observation that a less quantified decoction of savin leaves drunk or applied to the belly of a pregnant woman ‘destroyeth the living’ left too much to chance and appeared to be an invitation to abortion and murder and thus was not transcribed.^85^ Culpeper was maintaining a policy he first adopted in his translation of the *London Pharmacopoeia*, where, having inserted the uses of each of the herbal simples listed, declaimed ‘I willingly omitted the vertues of many of them, partly because I would not have the book too big, partly because they are not easily gotten, and many of the operations I buried in silence for fear knaves would put them in practice to do mischief’.^86^ In the past few decades, however, social historians of medicine have questioned such a position. John Riddle attributed the loss of oral knowledge passed from mother to daughter on how to regulate fertility to such a suppression of knowledge to promote large families or to control female sexual activity for religious or health reasons.^87^ It should be remembered, as well, that emmenogogic herbs that cause uterine contractions to stimulate menstruation had employment in female reproductive health as well as abortion.^88^ From a different standpoint, Mary Fissell saw in Culpeper’s *A Directory for Midwives* (1651) a negative implication for gender relations and midwifery education, where women can only learn from men like Culpeper and no longer rely on obtaining practical knowledge from each other on the use of such agents.^89^ Sanderson defended Culpeper by arguing that he deliberately excluded descriptions of practical procedures so that this knowledge could only be obtained through the midwives’ unofficial system of apprenticeship.^90^

Although the removal of a treatment indication might be warranted on safety grounds, other, more innocuous indications, such as the juice of brambles for heartburn, also went missing sometimes in the borrowing; whether it was from a need to economise on words more pressing in that moment, an oversight, or a lack of support for the specified use is not clear. Culpeper systematically omitted the galenic qualities of herbs, but some he retained for safety reasons, as a warning about herbs so hot they might burn, like garlic, or, with hemlock and the nightshades, so deadly cold they could kill.

Parkinson actually preserved much folklore of herbs in his text, touching a preoccupation of the period that saw a large number of pamphlets on folk medicine written in the sixteenth and seventeenth centuries.^91^ Both he and Culpeper discussed the truth of the deadliness of the ash tree to snakes, the belief obtained from watching swallows that the herb greater celandine healed human eyes also, or that concretions in their brains might control epileptic fits (Culpeper excised these himself once for an experiment he never completed) and the Christmas flowering of the Glastonbury thorn.^92^ Modern scholars date the rise of the concept of folklore to the mid-nineteenth century and identify its defining attributes as traditionalism, irrationality, and rurality, to be joined later by communality and universality.^93^ Keith Thomas referred to some seventeenth-century writers attempting to uncover ‘rational foundations’ to folk beliefs or superstitions but twentieth-century utilitarian, functional, and symbolic approaches to the problem fail to counter the argument that seventeenth-century England could not have made a new and coherent system out of the ‘cultural debris’ of preceding ages and thought.^94^ Parkinson scorned some contemporary beliefs but accepted others.^95^ Culpeper’s general engagement with this record of superstitions was to edit out unverified folk beliefs concerning plants, as he did with uses of herbs to engender lust or drunkenness or prevent conception, as objects of unworthy superstition and wrong practice and so more unnecessary information for his small herbal.^96^ He did, however, test out the truth of some superstitions and gave credence to the power of plants and stones used as amulets – although he never used this word in his writings – usually hung around the neck or otherwise held next to the body.^97^ For his part, Parkinson followed Dioscorides in seeking to maintain a rational explanation for the working of herbs and broadly rejected the use of amulets.

Considering the extent of Culpeper’s borrowing from Parkinson’s works, what is left that is original and from the author himself in *The English Physitian Enlarged*? He regularly inserted his own herbal knowledge among the entries, in simple uses of a particular herb, some of which have been described above, while others he instructed to be employed fresh in season as, for instance, the spring tonics tansy and dandelion for cleansing the body of ‘wintry humours’ and preparing it for a healthful change of season. Again, he might specify a simple way of preparing a herb as a medicine, and could refer the reader to the ‘Directions’ at the back of the book, where he taught how to gather and preserve the root, bark, leaf, flower, seed, or juice of any of the English herbs described, and how to make from them syrups and conserves, juleps and electuaries, pills, oils decoctions, and plasters.^98^ Herbs could be hung up to dry, kept in paper bags, and boiled into medicines in the kitchen in pots and pans, and sieved or layered fresh with sugar into jars. A mortar and pestle were needed to create powders out of dried herbs and the instructions on how to produce distilled waters from fresh, green plants required a pewter still. This kind of information supported domestic medicine without the need for a doctor, or indeed an apothecary like Parkinson.

Culpeper himself proposed three ‘profits and benefits’ of the book for the reader: by assigning a planetary ruler to each herb in an expression of Neoplatonic harmony between the celestial and elemental worlds, exemplifying the Hermetic axiom ‘as above so below’, so making appear ‘the infinite power and wisdom of god’; by conceding ignorance about the world after perusing the knowledge of herbal medicines laid out in the work; and by the illumination of its astrologo-physical explanations as the right way to begin a study of physic.^99^ The following year, he was able to direct readers of his new edition of the *Pharmacopoeia Londinensis* to the new catalogue of simples

…having published in print such a treatise of herbs and plants [*The English Physitian*] as my country men may readily make use of, for their own preservation of health or cure of diseases, such as grow near them and are easily to be had; that so by the help of my book they may cure themselves and never be beholding to such physitians as the iniquity of these times affords.

In these ways, Culpeper’s herbal differed greatly from old man Parkinson’s. His was by a ‘popular’ writer who supported the poor against the medical establishment, ‘making healing a political as well as a medical act’.^100^ His book sold for a few pence to a nation whose medical practitioners he accused of failing to serve their fellow countrymen.

## Conclusions

*The English Physitian* and its enlargement of the following year were highly successful publications for its author and his publisher which promoted the Culpeper name in print and his face through its frontispiece portrait. Its purpose was to help the sick who could not afford a physician, in other words most of the people of England, to know what might be good for their ailments from garden, field, and hedgerow, and this was achieved by making the information on English herbs contained in the country’s best herbal, Parkinson’s *Theatrum Botanicum*, available for three pence. It was part of a project to bring a ‘whol moddel of physick’ in the vernacular to the Commonwealth of England, to remove their dependence on physicians by promoting the right approach to the study of physic to achieve freedom of the individual in matters of health. By so doing, Culpeper ‘virtually reinvented’ vernacular medical publishing with a social purpose, following the multiple editions of bestselling health manuals of the sixteenth century, and many others followed him in the 1660s and 1670s with the claim also to be writing ‘for the public good’ and as fellow ‘students of physick and astrology’.^101^ One form of public good lay in the reform of English medicine, the possibility of health care for all being a cornerstone of Protestant ambition.

I have demonstrated in examples how Culpeper adapted Parkinson’s substantial works on herbs to his own more focused plans. This entailed the editing down of entries to remove what was superfluous to the communication of the medical uses of English herbs listed by Parkinson, to which Culpeper added some of his own. Most technical medical terms and non-medical and veterinary uses of plants were therefore omitted. The distilled waters of a few of the more dangerous native plants replaced the galenical preparations recorded by Parkinson – Culpeper’s cheap herbal would be perused by many less educated members of the reading public than might afford the other’s expensive folios, and so he needed to consider very seriously the safety of his readers – and even an alchemically prepared salt of chamomile was listed as the best option for kidney stones. With the majority of herbs Culpeper largely kept to galenical preparations because these were within reach of the poor physically and financially and could be turned into medicines without the need of equipment beyond what may be found in an ordinary kitchen. Warnings about herbs with abortifacient uses perhaps reflects Culpeper’s ethical practice as a would-be English physician, in distinction to Parkinson’s status as an apothecary who had been trained to dispense the prescriptions of learned members of the College of Physicians.

Botanical descriptions of less well-known herbs and specific plant locations were kept, but Culpeper’s frustration with his own attempts at morphological description suggests a lack of interest in the new science of plants. Culpeper was probably no more ignorant about plant identification than most apothecaries at the time, and he might have benefitted from instruction from apothecary and early botanist Thomas Johnson when he himself was in training. Moreover, his herbal did contain Parkinson’s detailed and careful descriptions for virtually all of the plants listed. Both authors raised folkloric and superstitious uses of plants, mainly to criticise such beliefs: Culpeper reported testing out the truth of some superstitions, whereas Parkinson appealed to reason and testimony of others in judging such beliefs. He also rejected the use of herbs as amulets while Culpeper promoted several examples of such application. Both authors made use to a limited extent of a doctrine of signatures, but Culpeper’s book was dominated by his astrologo-physical discourse that pointed the reader towards the appreciation of a harmony of creation that showed the wisdom and excellence of God. Wonders might be performed, he argued, with a herb rightly gathered under the influence of its planet.^102^ Parkinson was more conventional in both his religion and his science.

These revisions to Parkinson’s information on 328 plants, together with Culpeper’s own attractive and engaging style of writing, have led informed readers old and new to miss the extensive copying from one specific author in the creation of *The English Physitian Enlarged*, a process which has been analysed in this study.

## Footnotes

1

Nicholas Culpeper , A Physicall Directory (London: Peter Cole, 1649).

2

Elizabeth Lane Furdell , ‘”Reported to be distracted”: The suicide of Puritan entrepreneur Peter Cole’, The Historian, 66, 4 (2004), 772–792 .

3

Nicholas Culpeper , The English Physitian (London: Peter Cole, 1652), ‘authors made use of in this treatise’, B2v.

4

Richard Pulteney , Historical and Biographical Sketches of the Progress of Botany in England (London: Printed for T. Cadell, 1790), 180–1; Blanche Henrey , British Botanical and Horticultural Literature before 1800, Vol. I (London: Oxford University Press, 1975), 84 .

5

Agnes Arber , Herbals, their Origin and Evolution: A Chapter in the History of Botany 1470-1670 (Cambridge: Cambridge University Press, 1912), 212–20. For the meanings of the word ‘herbalist’, see Graeme Tobyn, ‘“Herbaries” and apothecaries: The identification of William Turner’s herbalists in Tudor and Stuart England’, *Vesalius*, XXIV,1, 67-75.

6 Arber, *op. cit.* (note 6), 144; Juanita Burnby, ‘*John Parkinson* (1567-1650)’, Oxford Dictionary of National Biography. www.oxforddnb.com.

7

Fabrizio Baldassarri (Ed.), Plants in 16th and 17th Centuries: Botany Between Medicine and Science (Berlin, De Gruyter, 2023), 4.

8

Eleanour Sinclair Rohde , The Old English Herbals (London: Dover Publications, 1922/1971), 163–167 ; Sarah Neville , Early Modern Herbals and the Book Trade: English Stationers and the Commodification of Botany (Cambridge: Cambridge University Press, 2021).

9

Frederick N.L. Poynter , ‘Nicholas Culpeper and his books’, Journal of the History of Medicine and Allied Sciences XVII, I, (1962), 152–167 10.1093/jhmas/xvii.1.15214037402; Nicholas Culpeper , An Ephemeris for the yeer 1651 (London: Peter Cole, 1651), A2r.

10 Rex F. Jones, ‘Genealogy of a Classic: The English Physitian of Nicholas Culpeper’, (unpublished PhD Thesis, University of California, San Francisco, 1984), iii; Jonathan Sanderson, ‘Nicholas Culpeper and the Book Trade: Print and the Promotion of Vernacular Medical Knowledge 1649-65’ (unpublished PhD thesis, University of Leeds, 1999), 141; John Parkinson , Theatrum Botanicum: The Theater of Plants, or An Herball of a Large Extent (London: Thomas Cotes, 1640).

11

Nicholas Culpeper , The English Physitian Enlarged (London: Peter Cole, 1653); John Parkinson*, Paradisi in Sole Paradisus Terrestris; or, A garden of all sorts of pleasant flowers which our English ayre will permit to be noursed up…collected by John Parkinson apothecary of London* (London, Humfrey Lownes and Robert Young at the signe of the Starre on Bread-street hill, 1629).

12

Nicholas Culpeper , A Physical Directory (London: Peter Cole, 1650), B2r.

13

Nicholas Culpeper , Galen’s Art of Physick (London: Peter Cole, 1652); Culpeper, *op. cit.* (note 9), K4r; Graeme Tobyn, ‘Did the astrological content of Culpeper’s *English Physitian* undermine its usefulness?’, *Culture and Cosmos*, 26, 2, Autumn/Winter 2022, 35-53.

14 Culpeper, *op. cit.* (note 11), C2v; Graeme Tobyn , ‘Dr Reason & Dr Experience: Culpeper’s assignation of planetary rulers in *The English Physitian*’, in Charles Burnett and Dorian Greenbaum (eds). From Masha’allah to Kepler: Theory and Practice in Medieval and Renaissance Astrology (Lampeter, Wales: Sophia Centre Press, 2015), 487–8; Sanderson, *op. cit.* (note 10), 145.

15 MeasuringWorth.com, *Purchasing Power of British Pounds from 1270 to Present*, https://www.measuringworth.com/calculators/ppoweruk/

16 Culpeper, *op. cit.* (note 11), 171.

17 Sanderson, *op. cit.* (note 10), 232, 236.

18 David E. Allen, ‘John Goodyer (1592-1664)’, Oxford Dictionary of National Biography, www.oxforddnb.com.

19 Peter Elmer, ‘The library of Dr John Webster: The making of a seventeenth-century radical’. Wellcome Institute for the History of Medicine: *Medical History*, Supplement No.6, items 2 & 666.

20

Elaine Leong , Recipes and Everyday Knowledge: Medicine, Science, and the Household in Early Modern England (Chicago: University of Chicago Press, 2018), 149 , 224 n.12.

21

Charles Webster , The Great Instauration; Science, Medicine and Reform 1626-60 (London: Duckworth, 1975), 267–71, 473.

22

Roy Porter , (1992) The popularisation of medicine 1650-1850 (London: Routledge, 1992), 2 .

23

Nicholas Culpeper , The English Physician (London: William Bentley, 1652) [BL 1606/2070]; On marginalia, see for instance Leong, *op. cit.* (note 20), 163.

24

William Coles , The Art of Simpling (London: Printed for Nathaniel Brook, 1656), 77 .

25

William Coles , Adam in Eden: Or, Nature’s Paradise (London: Printed by J. Streater for Nathaniel Brook, 1657), 158 .

26 Helen M. Dingwall, ‘Matthew Mackail (fl. 1657-96)’, Oxford Dictionary of National Biography, www.oxforddnb.com.; Matthew Mackaile , Moffet-Well; Or, a Topographico-Spagyricall Description of the Mineral Wells at Moffet…As, also at Oyly-Well…to these is subjoined A Character of Mr Culpeper and his Writings (Edinburgh: Printed for Robert Brown, 1664), 184 .

27

Olav Thulesius , Nicholas Culpeper English Physician and Astrologer (Basingstoke: The Macmillan Press, 1992), 105 ; Benjamin Woolley , The Herbalist: Nicholas Culpeper and the Fight for Medical Freedom (London: HarperCollins Publishers, 2004), 319 ; and my own *Culpeper’s Medicine* (Shaftesbury, Dorset: Element, 1997), 22-4. Thulesius’s study included fictitious episodes rendering it unreliable anyway ( Mary E. Fissell , Vernacular Bodies: The Politics of Reproduction in Early Modern England (Oxford, Oxford University Press, 2006), 136 , n.1); Woolley compared Culpeper’s work and writings with that of William Harvey; my book aimed to describe Culpeper’s presentation of Hippocratic-Galenic medicine with an astrological overlay.

28

Graeme Tobyn , ‘An anatomy of The English Physitian’, in Susan Francia and Anne Stobart (eds). Critical Approaches to the History of Western Herbal Medicine (London: Bloomsbury, 2014), 87–103 .

29

Clare J. Fowler , Pharmacopoeia Londinensis 1618 and its descendants. (London: Royal College of Physicians, 2018), 111 .

30 Tobyn, *op. cit.* (note 28), 93-6.

31 Arber, *op. cit.* (note 5), 115; Graeme Tobyn , Alison Denham and Margaret Whitelegg , The Western Herbal Tradition: 2000 Years of Medicinal Plant Knowledge (Edinburgh: Churchill Livingstone, 2011), 186 .

32

John Gerard , The herball: or, Generall Historie of Plantes. Gathered by John Gerarde of London, Master in Chirurgerie. Very much Enlarged and Amended by Thomas Iohnson Citizen and Apothecarye of London (London: Printed by Adam Islip, Ioice Norton, and Richard Whitakers, 1633), 825–6. This is now acknowledged as the earliest recorded sighting of this plant: https://plantatlas.brc.ac.uk/plant/stratiotes-aloides (accessed 5 May 2022).

33 Parkinson, *op. cit.* (note 10), 1249-50.

34

Adrian Johns , Piracy : The Intellectual Property Wars from Gutenberg to Gates (Chicago: University of Chicago Press, 2009), 83–108 .

35 John Gadbury, ‘The nativity of Nicholas Culpeper, student in physick and astrologer, calculated by John Gadbury’, in Nicholas Culpeper, *Culpeper’s School of Physick, or, The experimental practice of the whole art wherein are contained all inward diseases from the head to the foot, with their proper and effectuall cures, such diet set down as ought to be observed in sickness or in health* (London: printed for N. Brook, 1659), B2v-B8v, especially B8v.

36 Anon., ‘A life of the admired physician and astrologer of our times, Mr. Nicholas Culpeper’ in Culpeper, *op. cit.* (note 35), c1r. The Englished Dodoens text is Henry Lyte’s translation *A niewe herball, or historie of plantes* (London, Gerard Dewes, 1578).

37 Culpeper, *op. cit.* (note 11), 155, where the medicinal uses of sweet maudlin were left out ‘being the same with costmary or Alecost’ to which entry Culpeper referred the reader.

38 Culpeper, *op. cit.* (note 11), 110, in place of a description of the ‘furs-bush’ or gorse.

39 Parkinson, *op. cit.* (note 11), 39.

40 Culpeper, *op. cit.* (note 11), 13; Parkinson, *op. cit.* (note 11), 370.

41 Culpeper, *op. cit.* (note 11), 13.

42 Culpeper, *op. cit.* (note 11), 69, 77, 285.

43 Culpeper, *op. cit.* (note 11), C1r; Penelope Hunting , A History of the Society of Apothecaries (London: Worshipful Society of Apothecaries, 1998), 43–7.

44 Coles, *op. cit.* (note 24), 76-78. Coles was no doubt encouraged to be critical of Culpeper by his publisher Nathanial Brook, also the publisher of Culpeper’s astrological works, at a time when Brook’s struggle with Peter Cole over the Culpeper name was at its height. Graeme Tobyn , Culpeper’s Medicine, 2nd ed (London: singing Dragon, 2013), 27–33 .

45 Culpeper, *op. cit.* (note 11), 80; Parkinson, *op. cit.* (note 10), 324-43.

46 Culpeper, *op. cit.* (note 11), 97.

47

Anthony C. Cartwright , The British Pharmacopoeia, 1864 to 2014: Medicines, International Standards and the State (London: Routledge, 2015), 21 10.1080/00026980.2016.122718030463483.

48

John M. Riddle , Dioscorides on Pharmacy and Medicine. (Austin, University of Texas Press, 1985), 74–7.

49 Sanderson, *op. cit.* (note 10), 228, n. 106.

50

Andrew Wear , Knowledge and Practice in English Medicine 1550-1680 (Cambridge, Cambridge University Press, 2000), 98 .

51 Culpeper, *op. cit.* (note 11), 13-14, 60.

52

Gabrielle Hatfield , Memory, Wisdom and Healing: The History of Domestic Plant Medicine. (Thrupp, Glos.: Sutton Publishing, 2005), 5–7 ; Culpeper, *op. cit.* (note 11), 13.

53 Culpeper, *op. cit.* (note 11), 15, 375, 60.

54 L.G. Kelly, ‘Plato, Bacon and the Puritan Apothecary: The Case of Nicholas Culpeper’, *Target*, 1, 103.

55 Parkinson, *op. cit.* (note 10), 607; Culpeper, *op. cit.* (note 11), 15.

56 Culpeper, *op. cit.* (note 11), 87.

57 Culpeper, *op. cit.* (note 11), 362-63.

58 Michael Fothergill, C.T. Morgan, S. Jones, *et al.*, ‘Using Leaf-mark Material to Monitor the Morphology of White Clover (*Trifolium repens* L.) at the Clone and Ramet Level in Grazed Swards’, *Annals of Botany*, 88 (special issue), 797-802.

59 Culpeper, *op. cit.* (note 11), 80.

60 Woolley, *op. cit.* (note 27), 11.

61

Thomas Johnson , Iter Plantarum investigationis ergo susceptum a decem sociis in agrum Cantiarum…Ericetum Hamstediarum, sive plantarum ibi crescentium observatio (London?: A. Mathewes, 1629).

62 Culpeper, *op. cit.* (note 11), 69, 360.

63 Kelly, *op. cit.* (note 54), 103-4, 107

64 Culpeper, *op. cit.* (note 11), B3r.

65 Culpeper, *op. cit.* (note 11), 287; Parkinson, *op. cit.* (note 10), 728-9.

66 Parkinson, *op. cit.* (note 10), 934.

67 Culpeper, *op. cit.* (note 11), 123.

68 Parkinson, *op. cit.* (note 10), 553-54. The two sorts of Fluellen are *Kickxia spuria* and *K. elatine* (L.) Dumort. Kew Medicinal Plant Names Service: https://www.kew.org/science/our-science/science-services/medicinal-plant-names-services.

69 Culpeper, *op. cit.* (note 11), 108.

70 Culpeper, *op. cit.* (note 11), 108. Lluellin is written Llywelyn today.

71 Parkinson, *op. cit.* (note 10), 350; Culpeper, *op. cit.* (note 11), 2.

72 Parkinson, *op. cit.* (note 10), 693; Culpeper, *op. cit.* (note 11), 165.

73 Anon., *op. cit.* (note 36), C5v-C6r. For a reference to the use of an amanuensis for the writing of the herbal, see Culpeper, *op. cit.* (note 3), B1v.

74

Rebecca Laroche , Medical Authority and Englishwomen’s Herbal Texts 1550-1650 (Farnham: Ashgate, 2009), 60–1.

75

Margaret Pelling , ‘Compromised by Gender: The Role of the Male Medical Practitioner in Early Modern England’, in The Task of Healing: Medicine, Religion and Gender in England and the Netherlands (1485-1800), edited by Hilary Marland and Margaret Pelling (Rotterdam: Erasmus Publishing, 1996), 101–33.

76

Mary Fissell , ‘Introduction: Women, Health, and Healing in Early Modern Europe,’ Bulletin of the History of Medicine, 82, 1 (2008), 1–17 18344583 10.1353/bhm.2008.0024.

77 Culpeper, *op. cit.* (note 1), A2r.

78 A key study of such practices is David Gentilcore , Medical Charlatanism in Early Modern Italy (Oxford: Oxford University Press, 2006).

79

Paula De Vos , ‘Apothecaries, Artists, and Artisans: Early Industrial Material Culture in the Biological Old Regime’, The Journal of Interdisciplinary History, 45, 3, (2015), 277–336 , esp. 287; Susan Drury , ‘Herbal Remedies for Livestock in Seventeenth and Eighteenth Century England: Some Examples’, Folklore, 96, 2 (1985), 243–47.

80 Culpeper, *op. cit.* (note 11), 371.

81 Kelly, *op. cit.* (note 54), 99; Culpeper, *op. cit.* (note 1), B4r.

82 Anon., *The Epitaph* in Culpeper, *op. cit.* (note 36), c1v.

83 Culpeper used Parkinson’s words about a daily drink made from dandelion leaves to help those with ‘an evil disposition of the whol body, called a cachexia’, and for the same indication for chicory (then named succory) he strangely included the word printed in Greek letters as well as in English! Parkinson, *op. cit.* (note 10), 781; Culpeper, *op. cit.* (note 11), 84,349.

84 Culpeper, *op. cit.* (note 11), 335, 53; Culpeper, *op. cit.* (note 1), 152. Culpeper’s concern for the safe use of medicines will be reviewed in my forthcoming paper on versions of his translation of the London Pharmacopoeias of 1618 and 1650.

85 Parkinson, *op. cit.* (note 10), 1028.

86 Culpeper, *op. cit.* (note 1), 77.

87

John M. Riddle , Contraception and Abortion from the Ancient World to the Renaissance (Cambridge, MA: Harvard University Press, 157 ; Londa Schiebinger , Plants and Empire: Colonial Bioprospecting in the Atlantic World (Cambridge, MA: Harvard University Press, 2004), 119 .

88

Jennifer Evans , “Gentle Purges corrected with hot Spices, whether they work or not, do vehemently provoke Venery’: Menstrual Provocation and Procreation in Early Modern England’, Social History of Medicine, 25, 1 (2011), 2–19 ; Barbara Duden , The Woman Beneath the Skin: A Doctor’s Patients in Eighteenth-century Germany (Cambridge, MA: Harvard University Press, 1991), 104–178 .

89 Fissell, *op. cit.* (note 27), 135-156; Doreen Evenden , The Midwives of Seventeenth-century London (Cambridge: Cambridge University Press, 2000), 8–9 .

90 Sanderson, *op. cit.* (note 10), 195-7; Tobyn, *op. cit.* (note 44), 16. Fissell, *op. cit.* (note 27), 136 regretted being unable to consult Sanderson’s thesis.

91

Leslie F. Newman , ‘Some Notes on the Pharmacology and Therapeutic Value of Folk-Medicines I’, Folklore, 59, 3 (1948), 118–31.

92 Parkinson, *op. cit.* (note 10), 1419, 618, 1025; Culpeper, *op. cit.* (note 11), 21,76, 59, 122; Nicholas Culpeper, ‘The expert lapidary; or, A physical treatise of the secret vertues of stones’ in Culpeper, *op. cit.* (note 35), 274.

93

Dan Ben-Amos , ‘The idea of folklore’, in D. Ben-Amos (ed) Folklore concepts: Histories and critiques (Bloomington, Indiana University Press, 2020). Ben-Amos argued that while these attributes contributed to the popularity of folklore, they limited it as a field of scholarship for which he redefined it as a culturally unique mode of communication, formal, thematic, and performative.

94

Keith Thomas , Religion and the Decline of magic. (London: Weidenfeld & Nicholson, 1971), 625–28.

95 Rohde, *op. cit.* (note 8), 154.

96

Nicholas Culpeper , Pharmacopoeia Londinensis. (London: Peter Cole, 1653), 66, 144, 301 ; Anon., *The Epitaph* in Culpeper, *op. cit.* (note 36), c1r.

97 Culpeper, *op. cit.* (note 11), entries on Amara dulcis, Mistletoe, Peony and Vervain; Culpeper, *op. cit.* (note 1), 73-6.

98 Culpeper, *op. cit.* (note 11), 381-94.

99 Culpeper, *op. cit.* (note 11), C2r.

100

Mary Fissell , ‘Popular Medical Books’, in Joad Raymond , (ed) Oxford History of Popular Print Culture, vol 1: Beginnings to 1660 (Oxford: Oxford University Press, 2011): 418–31. Quote on p. 428.

101

Mary Fissell , ‘The Marketplace of Print’ in Mark R.S. Jenner and Patrick Wallis (eds), Medicine and the Market in England and its Colonies c.1450-1850 (London: Palgrave Macmillan, 2007), 108–32; Hugh G. Dick , ‘Students of physick and astrology: A survey of astrological medicine in the age of science’, Journal of the History of Medicine, 1 (1946), 300–15, 419-3310.1093/jhmas/1.3.41920994267.

102 Culpeper, *op. cit.* (note 11), 12.

